# Bias Amplification in Gender, Gender Identity, and Geographical Affiliation

**DOI:** 10.1021/acs.jcim.2c00533

**Published:** 2022-05-19

**Authors:** Michele Cascella, Thereza A. Soares

**Affiliations:** † Department of Chemistry and Hylleraas Centre for Quantum Molecular Sciences, University of Oslo, P. O. Box 1033, Blindern 0315, Oslo, Norway; ‡ Department of Chemistry, University of São Paulo 14040-901 Ribeirão Preto, São Paulo, Brazil

## Abstract

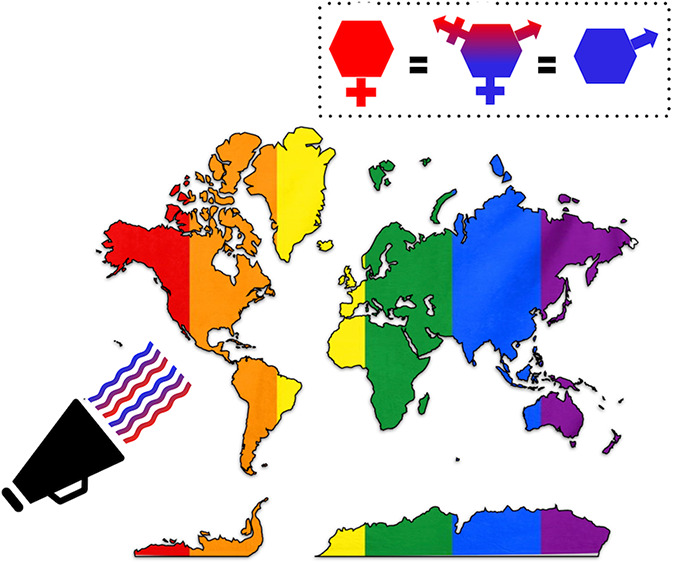

In the quest for greater equity in science, individual attitudes and institutional policies should also embrace greater diversity and inclusion of minority groups. This viewpoint calls for a broader definition of gender bias in STEM to include gender identity and for increased attention to the issue of bias amplification due to geographic affiliation in the field of computational chemistry and chemoinformatics. It briefly discusses some active interventions to tackle bias on gender, gender identity, and geographic affiliation in STEM.

## 1 Bias Amplification Based on Gender and Geographic Affiliation

It is accepted that diversity is critical to advance humanity’s scientific endeavors as it promotes better working groups^1^ and excellence in science.^2,3^ Yet, many groups still strive to “belong” in science. It has been 25 years since Wennerås and Wold’s pioneer study unveiled the occurrence of sexism and nepotism in grant allocation to postdoctoral researchers.^4^ In Sweden at that time, female scientists had to be ca. 2.5 times more productive than average male colleagues to receive the same competence score. Similar trends were soon verified in other countries (e.g., Denmark, Finland, UK, and USA),^5−7^ indicating that gender bias in grant peer review was a worldwide pattern. Since then, there has been continuous and concerted efforts within the scientific community and funding institutions to overcome gender-biased practices,^8−11^ even if some habits are hard to break.^12,13^ Despite important progresses toward gender equality in academic institutions, female representation in higher ranks and decision-making positions have not improved as fast or more significantly over the past 20 years.^9,14−,16^ Furthermore, it has become clear that gender bias in STEM is not a problem exclusive to women but to anyone who deviates from expected social stereotypes. It hits particularly hard the LGBTQIA+ and nonbinary people,^17,18^ and it may even affect white males who do not fit the presumed masculinity stereotype.^12,19,20^ In this viewpoint, we use the term gender in its most inclusive sense (e.g., female, LGBTQ+, nonbinary), although data on the status of nonheterosexual and/or noncisgender scientists remains scarce as many institutions, and funding agencies do not collect data on sexual orientation and gender identity.^21^

One factor of detrimental importance for research funding and career progression of scientists is the so-called “publication success”, which is often measured by the number and impact of publications and citations. Several reports have provided evidence of the unbalanced representation of females as authors in all investigated science fields.^4,16,18,22−26^ Chemistry was not an exception as shown by the assessment of each publication stage in chemical science journals from 2014 to 2018.^26^ Although one-third of the researchers in chemistry were female, female authorship percentage dwindled systematically throughout the stages of the publishing process with much lower success rates for female authors compared to male colleagues; the female percentage progressively decreased from first authors to corresponding authors to reviewers. The trend was more accentuated in higher impact factor journals. Moreover, papers with female corresponding authors, and to a lesser extent female first authors, had lower citation success than male corresponding authors. Female reviewers were also underrepresented, but rather because of low invitation frequency than a potential tendency to decline to review.^27^ In fact, it has been shown that female reviewers are more likely to assist with papers that have been under several cycles of revision.^26^ Although we do not have comparative data for nonheterosexual and noncisgender authors, we would expect an even lower representation of these groups in scientific publications.^17^

Another layer of complexity arises from the perception that publication success is influenced also by the geographic location of authors.^28−30^ Hence, the integrity of peer review has been questioned based on the evidence that outcomes can differ for authors in different countries.^31−33^ The existence and extension of biases based on author/reviewer nationality and prestige of institutional affiliation have been addressed with varying conclusions. On the one hand, some reports indicate that the income and development level of the origin country impact whether a manuscript is reviewed.^34^ Yet, this outcome may result from the fact that low-income country research can lack the quality (for different reasons, including poor or discontinuous funding) to meet publication criteria.^35^ On the other hand, other reports indicate the existence of systematic biases toward author/reviewer nationality and prestige of institutional affiliation. For instance, one experiment has shown that only one of nine articles originally published in a respected peer-review journal was accepted on resubmission to the same journal with the names of the original institutions changed to less prestigious ones.^36^ Another one indicates that manuscripts from outside North America were less likely to be accepted for publication.^37^ More recently, analysis of the demographics of authors, editors, peer reviewers, and peer-review outcomes for submissions to *eLife* have shown that male authors and authors affiliated with institutions in North America and Europe had greater publication success rates. These two groups were also over-represented among editors and reviewers pointing to the existence of reviewer homophily, i.e., the tendency of reviewers to be more favorable to papers by authors of the same gender or from the same country, in editorial decisions.^38^

It is clearly challenging to disentangle the contribution of peer review bias from multiple factors external to the review process (access to and continuity of funding, cultural factors, discipline, and individual abilities). Yet, this fact does not preclude the implementation of preemptive policies to combat gender and national disparities from bias in peer review. This is particularly important because in most computational chemistry publications only the identity of the reviewer is hidden (single-blind review). Although there is not sufficient data to conclude which model of peer review leads to the fairest and most impartial assessment, it is clear that authors are vulnerable to gender and social bias in a single-blind review model rather than a double-blind review model.^39^

## 2 Quest for Visibility by the LGBTQIA+

In a competition among poorly represented groups, women have an odd advantage with respect to other biased categories: their number and their intrinsic visibility. In a perfectly unbiased pool, women would constitute ∼50% of the ensemble; moreover, the male/female sex is commonly explicitly indicated in official forms. Consequently, it is relatively easy to perform statistical studies targeting the male/female dualism, as is witnessed by the conspicuous body of studies produced in the last few decades. Other biased categories suffer from the lack of such a systematic monitoring on their condition. This is detrimental for various reasons. Primarily, to tackle a problem, it must be first brought forward and recognized as such, and the only way to do it is by collecting hard statistical data. It is noticeable that the first widespread survey on bias and perception of LGBTQIA+ scientists in US academies has appeared as recently as only roughly one year ago.^39^ In this first large-case report, which involved a sample of over 25,000 employees in US universities, authors identified the presence of systematic potential inequalities for people belonging to the LGBTQIA+ spectrum in several professional aspects, including career opportunities, social exclusion, or health and wellness difficulties. Importantly, the study also pointed out that increased difficulties encountered during the professional life enhances the intention by this social group to leave current STEM jobs or to leave STEM entirely.^39^

To the best of our knowledge, no systematic scrutiny has ever been conducted in university systems in other areas of the world. This reflects a global socio-political panorama where the LGBTQIA+ community is still openly discriminated or directly prosecuted in several countries, making such investigations practically unfeasible, with even the potential of harming people contributing to it. Remarkably, even in Europe, where several countries are at the forefront of legal recognition of LGBTQIA+ issues, there is no national legislation that fully levels the rights of LGBTQIA+ citizens to those of the others.^40^ Second, and most importantly, the lack of visibility eases the chance that governmental equal opportunity policies in different countries are steered toward overlooking the issues of these categories, promoting instead a cultural environment that keeps its focus elsewhere. It has been reported that LGBTQIA+ STEM professionals are less likely than their peers to whistleblowing.^40^ This is likely due to generalized mistrust in a system being able to appropriately recognize and consider their issues and rights. The generalized mistrust in the system by the LGBTQIA+ community in academia has recently been confirmed in a study centered in southwestern US universities.^40^ This study highlighted that LGBTQIA+ college and university students less likely report bias incidents to campus or legal authorities than peers, evidencing a more generalized discomfort by this social group in living in the academic environment. This particularly worrisome, considering that several studies show that LGBTQIA+ students are statistically more exposed to sexual harassment and assault.^41,42^

## 3 Recommendations to Promote Equity of Gender, Gender Identity, and Geographical

Fighting bias is a complicated issue, because the environment in which it occurs is often not the one that has primarily created it. Ultimately, lifting bias in working places would be fully achieved by promoting more fundamental changes into the societal structure itself. Discussing policies that can be effective in promoting such cultural reforms falls well beyond the scope of the present text. Here, we just notice how the problem is of extreme complexity, pointing to the fact that even Nordic countries, commonly referred to as the most advanced in promoting gender equality, have not fully overcome this issue.^43^ Yet, first and foremost, it is important to recognize that implicit and unconscious bias affects us all, requiring a deliberate and continuous effort to be deterred at the individual and organization levels. Fortunately, there is evidence that learning about unconscious bias increases individual willingness to acknowledge ones own susceptibility to it.^44^ Organizations should continuously promote implicit bias awareness as a means to ensure the fair assessment of minority groups. In this regard, a total of 52 academic publishers with a portfolio of more than 15,000 journals, including ACS, have agreed on a framework for action to reducing bias and continuously scrutinize their publication processes to ensure a more inclusive and diverse culture within scholarly publishing. The *Joint Commitment for Action on Inclusion and Diversity in Publishing* group have further pledged to collect self-reported information on the demographic diversity (gender identity, race, and ethnicity) of authors, editorial decision makers, and reviewers. This is an important step to accurately define where bias lies in scholarly publishing and to craft more efficient policies to fight it. Several measures such as training of reviewers, making public policies on conflict of interest, and providing reviewers with clear guidelines on the evaluation criteria have also been shown to be highly effective to prevent or limit bias in grant allocation by funding agencies.^45,46^

In the opinion of the authors, the primary difficulty encountered by people belonging to minority groups is associated with lack of representation and visibility. This has the potential to create conditions leading to several forms of bias that can synergize in negative feedback loops. For example, a lack of representation may induce bias toward default expectations for types of names or personalities. On the other hand, the same persons that fall out of default schemes are more easily prone to develop “impostor syndrome” feelings. Changing this attitude requires direct initiatives aimed at bringing forward the existence and the positive contributions by all those scientists belonging to minority groups at large. Geographical bias can be challenged by initiatives aimed at promoting the participation of the global scientific community as a whole. Here, funding agencies can play a direct role, for example, by increasing funding for international collaboration between rich and developing countries. Major global conferences should facilitate the participation of scientists from all parts of the world, especially as speakers, also by offering reduced fees or helping with travel grants. In this regard, a successful initiative funded by the Research Council of Norway is the conference series FemEx: *Female Excellence Theoretical and Computational Chemistry*, in which women comprised 80% of the keynote speakers.^47^ The three international meetings held so far offer a bold statement that there is no shortage of highly qualified female scientists in all fields of theoretical and computational chemistry to act as speakers in conferences. Similar initiatives directed to the LGBTQIA+ scientific community will bring greater visibility, facilitate networking, and strengthen identities and the sense of “belonging”.^48^

Regional differences affect even more dramatically the visibility and acceptance of the LGBTQIA+ community. Nonetheless, at a global level, the LGBTQIA+ STEM community shares the problem of poor monitoring compared to other biased groups. There is thus an urgent need of establishing systematic statistical studies aimed at tracking the extent and evolution of bias in order to implement the most appropriate policies to face this issue. Visibility for minorities has the potential to create positive role models that give the strength of other people to feel they can be part of the academic system. In this respect, we welcome editorial initiatives like the recent special article collection in *Inorganic Chemistry* curated by Ghosh and Tolman, which brought forward major scientific contributions in the field by authors who identify themselves in the queer spectrum.^49^ As curators point out in their introductory editorial: *“[..] Much of the discussion around identity-based discrimination in the West has focused on either systemic or institutional bias or the most scandalous and grotesque forms of harassment. However, much damage is also done by day-to-day obstruction and milder forms of harassment, which over time can exact a devastating toll.”*(49) The role of local institutions in fighting such subtle forms of discrimination and bias is crucial. As pointed out before, statistics report that minority groups have less tendency to whistleblowing. Thus, just establishing rules for equal opportunity and relative offices is not sufficient. On the contrary, inclusion policies must be raised at a proactive level, directly promoting the visibility of minority groups. Also, it is necessary that all personnel are appropriately educated on most widespread inclusion policies.

In conclusion, this commentary calls for a broader definition of gender bias to include gender identity in STEM and for greater attention to the issue of bias amplification due to geographic affiliation in the field of computational chemistry and chemoinformatics. The pathways to greater equity, diversity, and inclusion have already been addressed by several studies.^29,33,39,46,47,49−52^ One common outcome from these reports is the need for organizations to actively seek and promote greater gender identity and geographical diversity on their hiring bodies, evaluation panels, and editorial boards. Another is the need for individuals to act proactively to recognize and counteract implicit or explicit bias. Together, as a community, we must engage ourselves to fight bias, promoting greater equality and excellence in computational chemistry and chemoinformatics for all.
